# Premenstrual syndrome as a possible presymtomatic marker for negative outcomes of pregnancy

**DOI:** 10.1192/j.eurpsy.2022.299

**Published:** 2022-09-01

**Authors:** E. Eros

**Affiliations:** Health and Well-being Clinic, Psychiatry, Budapest, Hungary

**Keywords:** premenstrual syndrome, prevention, post natal depression

## Abstract

**Introduction:**

Hungarian Family Planning Service’s mission is decreasing the negative outcomes of pregnancy, including perinatal depression.

**Objectives:**

Childbirth as a great irreversible life event is a normative crisis of the life, thus pregnancy and post-partum period are times of high risk for psychiatric symptoms. Stress in pre- and post-natal period has short and long-term effect on offspring. Women participating in family planning program should be evaluated for the high risk and specific preventive program are provided for them.

**Methods:**

Between 2015-2018, 446 women were participating in family planning service. They were screened for premenstrual syndrome by using the shortened form of PAF questionnaire. We compared healthy and PMS affected patients’ data in according to the prevalence of PPD, spontaneous abortion and period needed for conception.

**Results:**

Prevalence of PMS in our sample was 51.4%. Overage duration between wished and realized conception was 6.1 months in healthy group vs 9.2 months in PMS group. Post-natal depression was screened by Edinburgh Post-natal Scale and it showed about 4-times higher prevalence between affected women by PMS. Surprising the rate of spontaneous abortion was 2-times higher, although the absolute number is rather low for statistical validation.

**Conclusions:**

Women affected by PMS can be considered as high risk for perinatal mood disorders and negative outcomes of pregnancy. PMS can be useful as a presymptomatic marker of perinatal depression and may be increased risk for spontaneous abortion. Psychological aspect should be included into the periconceptional care. Family planning may be an optimal solution to prevent perinatal depression and its complication.

**Disclosure:**

No significant relationships.

